# *Loki zupa (Luooukezupa)* decoction reduced airway inflammation in an OVA-induced asthma mouse model

**DOI:** 10.1186/s13020-016-0094-9

**Published:** 2016-04-29

**Authors:** Ying Wei, Muhammadjan Abduwaki, Mihui Li, Qingli Luo, Jing Sun, Yubao Lv, Mammat Nurahmat, Jingcheng Dong

**Affiliations:** Department of Integrative Medicine, Huashan Hospital, Fudan University, 12 Middle Urumqi Road, Shanghai, 200040 China; Institute of Integrated Traditional Chinese and Western Medicine, Fudan University, 12 Middle Urumqi Road, Shanghai, 200040 China; Xinjiang Uygur Medical College, 370 West Beijing Road, Hotan, Xinjiang, 848000 China

## Abstract

**Background:**

*Loki zupa* (*Luooukezupa*) decoction, consisting of the roots of *Hyssopuscuspidatus Boriss* (*Shenxiangcao*) and *Irishalophila Pall* root (*Yuanweigen*), is commonly used in Uygur medicine to treat asthma. However, the mode of action of this material has yet to be elucidated. This study aims to investigate the effects of *Loki zupa* decoction on the airway inflammation of an ovalbumin (OVA)-induced asthma mouse model.

**Methods:**

Mice were divided into normal control (NC), asthma (A), high, medium and low doses of *Loki zupa* decoction (L 14.0, L 7.0, L 3.5), water extract (LW), *n*-butanol extract (LN), ethyl acetate extract (LE) and dexamethasone (DEX) groups. Antiasthmatic model was induced by OVA sensitization and challenged using BALB/c mice. Airway hyperresponsiveness (AHR) toward methacholine (Mch) was assessed using Buxco equipment. Lung inflammation was measured by hematoxylin and eosin staining and bronchoalveolar lavage fluid (BALF) cell count and classification. Inflammatory cytokines in BALF and serum were analyzed by Bio-Plex assay, and mRNA levels were investigated by qPCR analysis. The roots of *H. Boriss* (250 g) and *I. Pall* (250 g) were decocted, concentrated and diluted to 14.0, 7.0 and 3.5 g crude herb/kg body weight. The LW, LN and LE of the *Loki zupa* decoction were prepared and diluted to a dose equivalent to 7 g of crude herb/kg body weight.

**Results:**

*Loki zupa* decoction and its extracts significantly attenuated the AHR towards Mch (all *P* < 0.05). Treatment with *Loki zupa* decoction and its extracts relieved the infiltration of inflammatory cells in and around the airways, and reduced the total white blood cell (all *P* < 0.05), neutrophil (all *P* < 0.05), monocyte (all *P* < 0.05) and eosinophil (all *P* < 0.05) counts in the BALF. The BALF samples collected from the mice treated with the *Loki zupa* decoction and its extracts had lower levels of IL-1β (all *P* < 0.05), TNF-α (all *P* < 0.05), IL-2 (all *P* < 0.05), IL-4 (*P* = 0.047) and IL-5 (all *P* < 0.05). The serum samples of these mice also had lower IL-1β (all *P* < 0.05), TNF-α (all *P* < 0.05), IL-4 (all *P* < 0.05) and IL-5 (all *P* < 0.05) levels and higher levels of IFN-γ (*P* < 0.001) compared with the OVA-induced asthma mouse model. qPCR analysis revealed that *Loki zupa* decoction and its extracts inhibited mRNA expression of IL-4 (all *P* < 0.05), IL-5 (all *P* < 0.05) and IL-13 (all *P* < 0.05) and promoted mRNA expression of IFN-γ (all *P* < 0.05) in asthmatic mice.

**Conclusion:**

*Loki zupa* decoction reduced AHR, attenuated airway inflammation, promoted Th1 and suppressed Th2 cell functions in an OVA-induced asthma mouse model.

**Electronic supplementary material:**

The online version of this article (doi:10.1186/s13020-016-0094-9) contains supplementary material, which is available to authorized users.

## Background

Asthma is one of the most prevalent respiratory diseases in the world, and the prevalence of this disease is increasing on an annual basis [1]. Asthma is characterized by the inflammation of the airways, with the airways also becoming hyperresponsive. Furthermore, the inflammatory cells can infiltrate the airways, resulting in the secretion of related pro-inflammatory mediators [2]. Inhaled corticosteroids are currently the most effective drugs available for the treatment of asthma [3, 4]. However, the use of these drugs is often accompanied by undesirable side effects.

The Th2-driven inflammation of the eosinophilic airways is responsible for upto 50 % of all cases of asthma, and is generally considered to be the major pathogenetic factor in this disease [4–6]. Asthmatic patients typically have elevated levels of Th2 cytokines in their BALF and bronchial mucosa [7]. It is well known that Th2-activated cells orchestrate pulmonary immune responses and mediate the inflammation of lung tissues, as well as airway hyperresponsiveness [8–10]. Th1-activated cells also inhibit the inflammation of asthmatic airways [11]. It is noteworthy that mice deficient in the Th1-type transcription factor T-bet develop spontaneous AHR and augmented airway eosinophilia [12]. The immunomodulation of the Th1/Th2 imbalance encountered in asthma patients is therefore considered to be a practical strategy for controlling asthma [11].

*Loki zupa* (*Luooukezupa*) decoction, which consists of a 1:1 mixture (w/w) of the roots of *Hyssopuscuspidatus Boriss* (*Shenxiangcao*) and *Irishalophila Pall* (*Yuanweigen*), is commonly used in Uygur medicine for the treatment of asthma [13, 14]. Although the oral administration of *Loki zupa* decoction alleviates asthmatic symptoms, the exact mode of action of this drug has not been elucidated.

This study aims to investigate the effects of *Loki zupa* decoction on the inflammation of the airways in an ovalbumin (OVA)-induced asthma mouse model.

## Methods

### Animals

Female BALB/c mice (6 weeks old) were purchased from Shanghai Sippr BK Laboratory Animal Co., Ltd (Shanghai, China) and raised in a pathogen-free rodent facility according to the procedures approved (2013-06-HSYY-DJC-02; Additional file 1) by the Committee on the Ethics of Animal Experiments of Fudan University. The mice were housed in separate stainless steel cages (six mice per cage) in a temperature-controlled environment (20–24 °C) on 12 h light–dark cycles with unrestricted access to food and water. All procedures of this study were in accordance with “Experimental Animal Ordinance” of Fudan University. The animal studies were performed following the ARRIVE guideline (Additional file 2).

### Reagents

**Fig. 1:**
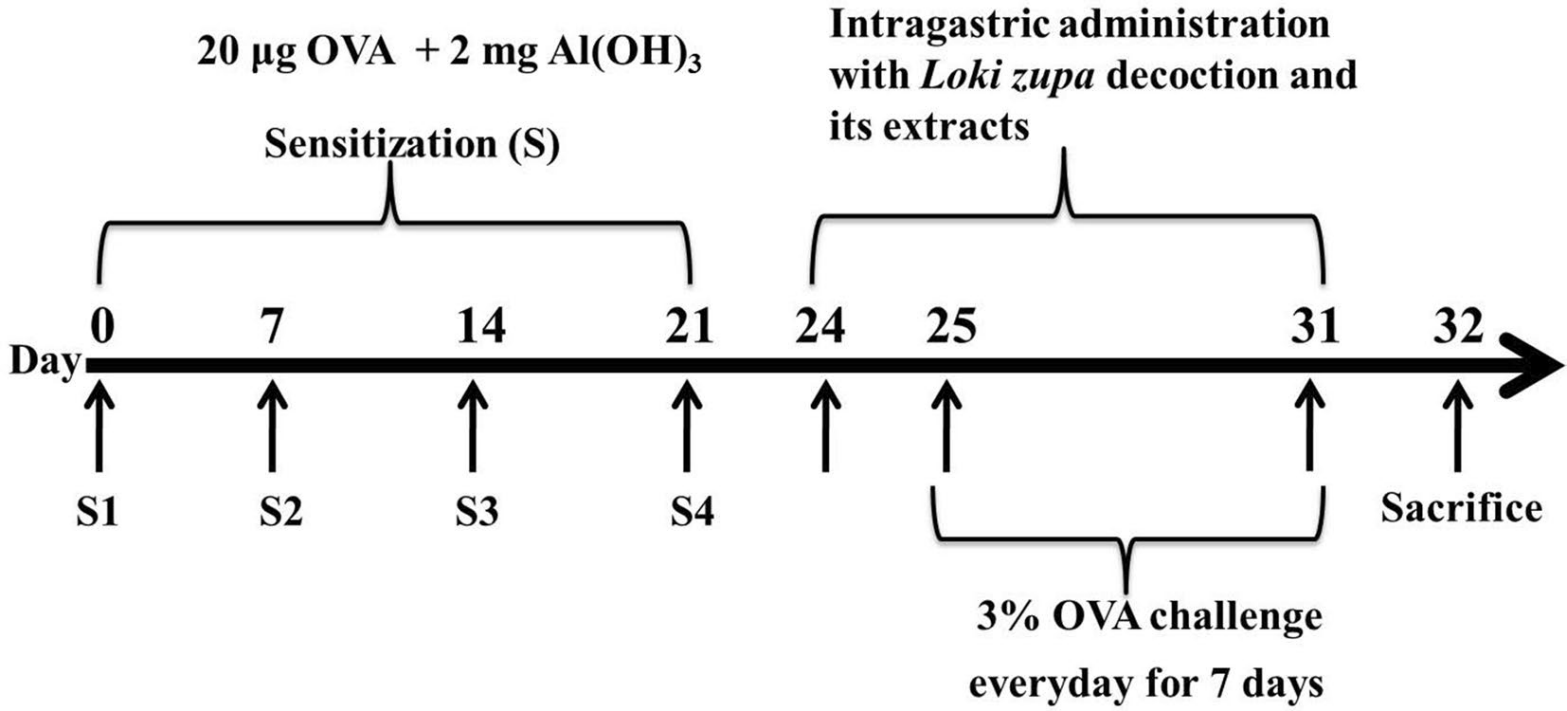
Experimental protocol for the OVA-induced asthmatic model and treatment processes

OVA and Mch were purchased from Sigma-Aldrich (St. Louis, MO, USA).The Bio-Plex kit used in this study was purchased from Bio-Rad (Hercules, CA, USA). The roots of *H. Boriss* and *I. Pall* were provided by the Xinjiang Uygur Medical College Affiliated Hospital, China. Small samples of these herbs were identified [15–17] by experts at the Testing Center of Jiangyin Tianjiang Pharmaceutical Co., Ltd (Jiangyin, China), where samples were also deposited with the following voucher specimen numbers: LZ-2013-01 to 02. Dexamethasone (DEX) was purchased from Chenxin pharmaceutical Co. Ltd (Jining, China) (Fig 1).

### Preparation and extraction of a *Loki zupa* decoction

The roots of *H. Boriss* (250 g) and *I. Pall* (250 g) were decocted in water (3.6 L) for 1 h (two times). The decoction was then filtered through a sterile gauze and concentrated to 1000 mL (equivalent to 500 g of crude herb per 1000 mL of decoction) using a concentration heater. The concentrated decoction was subsequently diluted to several different concentrations (i.e., 14.0, 7.0 and 3.5 g of crude herb/kg body of weight) before being used.

For the extraction of the *Loki zupa* decoction, the roots of *H. Boriss* (1250 g) and *I. Pall* (1250 g) were ground into a fine powder using a pestle and mortar. The resulting powder was degreased with petroleum ether and extracted three times with 70 % ethanol. The combined extracts were then filtered through sterile gauze and concentrated under vacuum on a rotary evaporator (EYELAN-1000, EYELA, Tokyo, Japan). The concentrated solution (predominantly aqueous) was extracted sequentially with petroleum ether, *n*-butanol and ethyl acetate. The water (LW), *n*-butanol (LN) and ethyl acetate (LE) extracts of the *Loki zupa* decoction were diluted to a concentration equivalent to 7 g of crude herb/kg body weight before being used.

### Asthmatic model establishment and treatment

Female BALB/c mice (n = 108) were arbitrarily divided into nine groups (n = 12 each group), including normal control (NC), asthma (A), *Loki zupa* decoction (L 14.0, L 7.0, L 3.5), LW, LN, LE and DEX groups. As shown in Fig. 1, mice in group A and the other treatment groups were sensitized with an intraperitoneal injection of 0.2 mL saline containing 20 μg OVA and 2 mg aluminum hydroxide on days 0, 7, 14 and 21. From day 25, the mice in group A and the other treatment groups were challenged for 7 consecutive days with a 3 % (w/v) OVA solution, which was administered using an ultrasonic nebulizer (402AI ultrasonic nebulizer, Yuyue, China). The mice in the NC group were sensitized and challenged with saline. From day 24, the mice in the NC and A groups were intragastrically administrated with saline, whereas the other treatment groups were administrated with 14.0, 7.0 and 3.5 g/kg *Loki zupa* decoctions and its extracts, as well as DEX.

### Measurement of AHR to Mch

Twenty-four hours after their last OVA challenge, the mice were anesthetized with pentobarbital sodium (50 mg/kg) to allow for their AHR to be measured using an invasive approach, as described previously, using Buxco resistance and compliance measurements (FinePointe RC System, Buxco, Wilmington, NC, USA) [18]. Increasing doses of Mch (0, 3.125, 6.25 and 12.5 mg/mL) were inhaled by nebulization to allow for the measurement of the AHR of the mice. Changes in airway resistance (R_L_) and lung dynamic compliance (Cdyn) were measured and these data were subsequently expressed as a percent change from the baseline value.

### Blood and bronchoalveolar lavage fluid (BALF) collection

Blood samples were collected from the mice immediately after the AHR measurements and stored at 4 °C for 2 h before being centrifuged at 5000×*g* for 15 min at 4 °C (5804R, Eppendorf, Leipzig, Germany). The resulting serum was collected, repackaged and stored at –80 °C prior to being analyzed in a Bio-Plex assay (Bio-Plex 200 System, Bio-Rad, Hercules, CA, USA). BALF collection was performed by lavaging the left lung with 0.3 mL aliquots of PBS (two times) through the tracheal cannula (total volume 0.6 mL). The resulting mixture was centrifuged at 1000×*g* for 10 min at 4 °C. The supernatant was collected and stored at –80 °C for further assay. The pellet from the BALF was resuspended in PBS (0.1 mL) for cell count and classification analysis using an automatic hematology analyzer (BC-5300 Vet, Mindray, China).

### Pulmonary histopathology analysis

Samples of the lung tissues were collected, fixed in 4 % paraformaldehyde, embedded in paraffin and cut into 5 μm sections for histopathological analysis. Lung sections were stained with hematoxylin and eosin (H&E) to evaluate any inflammatory changes. Ten mice from each group and six arbitrarily selected fields in each mouse were photographed using an optical microscope (ECLIPSE 80i, Nikon, Japan) and the resulting images were analyzed in detail. The severity of any inflammatory cell infiltration in the lung samples was evaluated using a 5 point scoring system: 1, no cells; 2, a few cells; 3, a ring of cells with a depth of one-cell layer; 4, a ring of cells with a depth of 2–4 cells; and 5, a ring of cells with a depth of >4 cells. The degree of airway inflammation in each group was determined by two independent analysts. If their results were different, a third analyst was considered for further analysis of the related data.

### Inflammatory mediators assay in BALF and serum

Cytokines present in the BALF and serum, including IL-1β, TNF-ɑ, IFN-γ, IL-2, IL-4 and IL-5 were assayed by Bio-Plex according to the manufacturer’s instructions.

### Real-time quantitative PCR (qPCR) analysis

qPCR analysis of the cytokine expression levels in the lung samples collected from the mice was performed according to the manufacturer’s instructions. The total RNA isolated from the lung tissue in each group was reverse transcripted into the first strand of cDNA using the Trizol reagent and a RevertAid TM First Strand cDNA Synthesis Kit (Fermentas, Vilnius, Republic of Lithuania). The qPCR amplification experiments were subsequently performed according to the manufacturer’s instructions using a PCR Amplifier (ViiA7, life technologies, Carlsbad, CA, USA). The final data were normalized to determine the level of GAPDH expression in each group, and the results were presented as fold changes compared with the expression levelsin mice belonging to the NC group. The primers’ sequences used for IL-4, IL-5, IL-13, IFN-γ and GAPDH are shown in Table 1.

**Table 1:** Primer sequences used for qPCR amplification

Gene	Primer sequence
IL-4 (NM_021283.2)	Forward primer: 5′-CAGCAACGAAGAACACCACA-3′ Reverse primer: 5′-AATCCAGGCATCGAAAAGCC-3′
IL-5 (NM_010558.1)	Forward primer: 5′-CAGTGTGAATGAGAGCCAGC-3′ Reverse primer: 5′-TTCTGTTGGCATGGGGTAGT-3′
IL-13 (NM_008355.3)	Forward primer: 5′-TTGAGGAGCTGAGCAACA-3′ Reverse primer: 5′-CCATGCTGCCGTTGCA-3′
IFN-γ (NM_008337.3)	Forward primer: 5′-AGCTCTTCCTCATGGCTGTT-3′ Reverse primer: 5′-TCCTTTTGCCAGTTCCTCCA-3′
GAPDH (NM_008084.2)	Forward primer: 5′-GTGGAGTCTACTGGTGTCTTC-3′ Reverse primer: 5′-CTCAGTGTCCCACCACCCT-3′

### Statistical analysis

Data were expressed as the mean ± standard deviation (SD). SPSS 19.0 for Windows (IBM, Chicago, Illinois, USA) was used to analyze the significance of the differences between the different groups, using 10 mice from each group. Statistical analysis of these data was performed by one-way analysis of variance (ANOVA) for multiple comparisons followed by the least significantly different or Games-Howell test for a comparison of the different groups. *P* values less than 0.05 were considered statistically significant.

## Results

### *Loki zupa* decoction and its extracts treatment decreased AHR of OVA-induced asthmatic mice

**Fig. 2:**
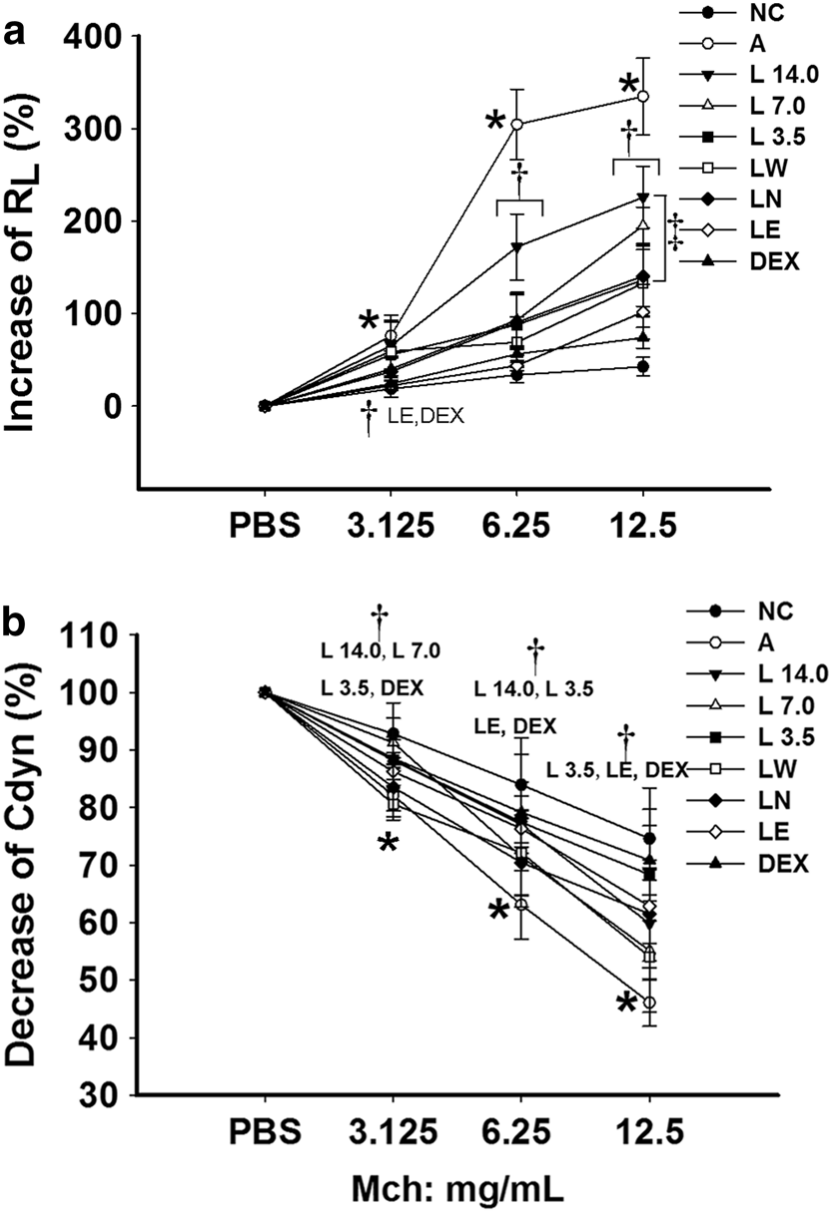
*Loki zupa* decoction and its extracts decreased AHR in the OVA-induced asthmatic mice compared with the untreated mice. AHR towards progressive doses of Mch was measured by an invasive approach 24 h after the last OVA challenge. Changes in R_L_ and Cdyn have been expressed as percentage changes from the corresponding baseline values. The abbreviations for NC, A, L, LW, LN, LE, DEX are as follows: *NC* normal control, *A* asthma, *L 14.0, L 7.0, L 3.5 Loki zupa* decoction, *LW* water extract, *LN* *n*-butanol extract, *LE* ethyl acetate extract, *DEX* dexamethasone. *P < 0.05 *vs.* NC group, ^†^ *P* < 0.05 *vs.* A group, ^‡^ *P* < 0.05 *vs.* DEX group

The results of this study showed that the short-term OVA challenge of mice with Mch led to a significant decrease in AHR in the R_L_ group compared with mice in the NC group (*P* = 0.037, *P* = 0.003, *P* = 0.001; Fig. 2a). Treatment of the mice with *Loki zupa* decoction or its extracts resulted in a significant decrease in the AHR of the R_L_ group towards Mch compared with the OVA-induced asthmatic mice (*P* < 0.05; Fig. 2a). Furthermore, the treatment of the mice with *Loki zupa* decoction and its extracts led to an obvious increase in their inhibitory effects towards Mch in the R_L_ group. For example, at a Mch dose of 3.125 mg/mL, LE and DEX demonstrated significant inhibitory activity towards the R_L_ group (*P* = 0.041, *P* = 0.047; Fig. 2a). Furthermore, all of the *Loki zupa*-and DEX-treated groupsled to a considerable decreasein AHR in the R_L_groups at a Mch dose of 6.25 mg/mL compared with OVA-induced asthmatic mice (*P* = 0.001, *P* < 0.001, *P* < 0.001, *P* < 0.001, *P* < 0.001, *P* < 0.001, *P* < 0.001). At aMch dose of 12.5 mg/mL, all of the treatment groups resulted in a significant decrease in AHR in the R_L_ group compared with the asthmatic mice (*P* = 0.002, *P* < 0.001, *P* < 0.001, *P* < 0.001, *P* < 0.001, *P* < 0.001, *P* < 0.001; Fig. 2a). The L 3.5 and LE-treated groups showed the greatest inhibitory effect of all of the groups tested in the current study towards AHR in the R_L_ group, which were the same as that of DEX at a Mch dose of 12.5 mg/mL (*P* = 0.364, *P* = 0.362; Fig. 2a).

Short-term OVA challenge in the mice also led to a significant reduction in Cdyn towards progressive doses of Mch compared with the mice in the NC group (*P* = 0.021, *P* = 0.002, *P* < 0.001; Fig. 2b). Treatment with *Loki zupa* decoction or its extracts resulted in improved Cdyn compared with the OVA-induced asthmatic mice. Furthermore, DEX treatment led to a dramatic increase in Cdyn compared with the asthmatic mice (*P* = 0.035, *P* = 0.004, *P* < 0.001). Different doses of *Loki zupa* decoction and its extracts resulted in a diverse range of effects on Cdyn to progressive doses of Mch. At a Mch dose of 3.125 mg/mL, all three doses of the *Loki zupa* decoction treatment tested in the current study led to significant increases in Cdyn (*P* = 0.031, *P* = 0.027, *P* = 0.032) compared with the asthmatic mice, representing a similar effect to that of DEX (*P* = 0.871, *P* = 0.329, *P* = 0.877). At a Mch dose of 6.25 mg/mL, the L 14.0 and L 3.5 treatment groups, as well as the LE treatment group, showed significant increases in Cdyn (*P* = 0.010, *P* = 0.012, *P* = 0.017) compared with the asthmatic mice, which were similarto the effect of DEX (*P* = 0.630, *P* = 0.633, *P* = 0.513). At a Mch dose of 12.5 mg/mL, only the L 3.5 and LE treatment groups showed significant increases in Cdyn (*P* = 0.003, *P* = 0.007) compared with asthmatic mice, with DEX treatment resulting in no significant changes (*P* = 0.693, *P* = 0.217).

### Treatment with the *Loki zupa* decoction and its extracts attenuated pulmonary inflammation in the OVA-induced asthmatic mice

**Fig. 3:**
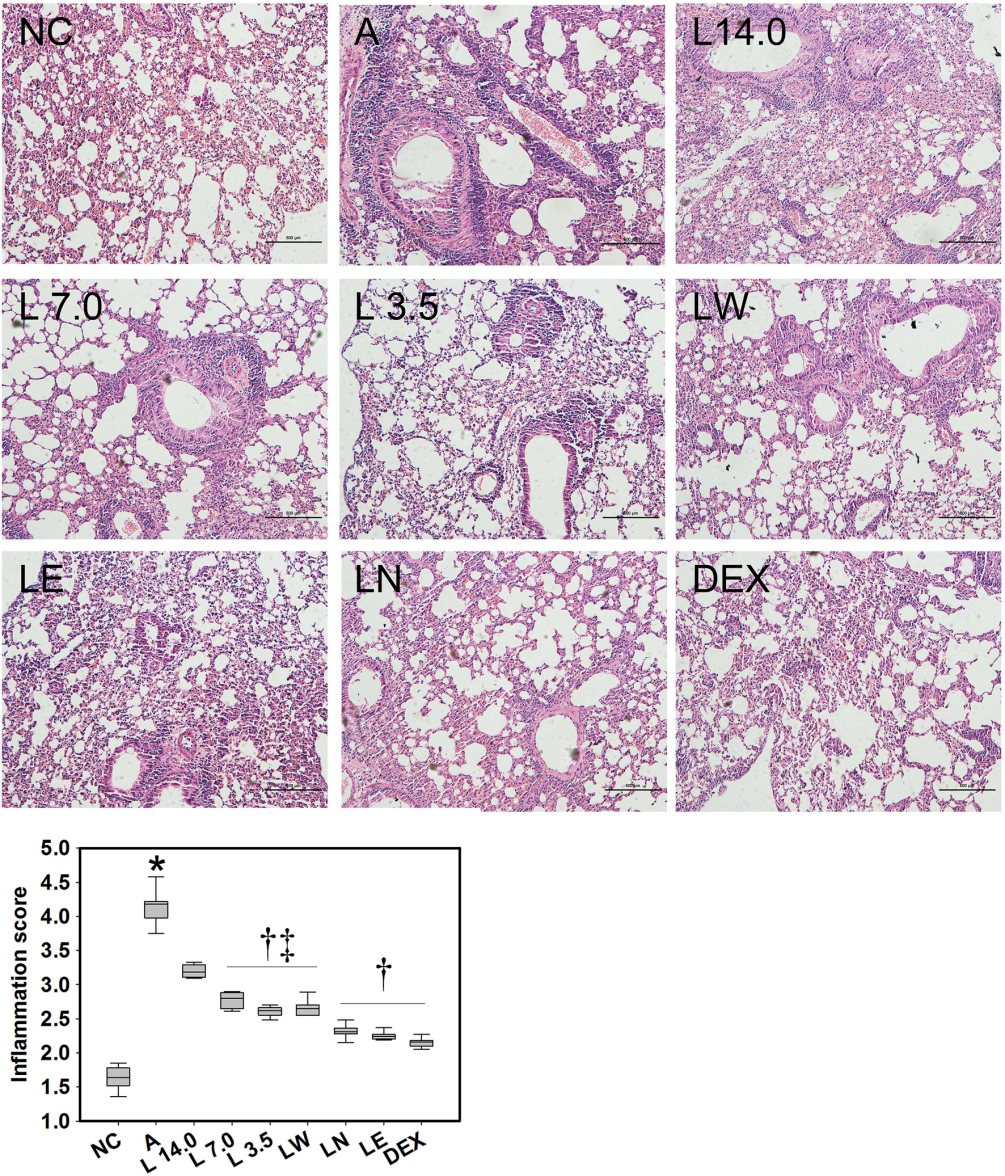
*Loki zupa* decoction and its extracts attenuated airway inflammation of OVA-induced asthmatic mice. The airway inflammatory changes in each group were assessed by H&E staining of the lung slices and examined at 10× magnification by light microscopy. At least ten mice in each group and six arbitrarily selected fields in each mouse were assessed. Inflammatory scores in each group were assessed. Bar = 500 μm. *P < 0.05 *vs.* NC group, ^†^ *P* < 0.05 *vs.* A group, ^‡^ *P* < 0.05 *vs.* DEX group

Short-term OVA challenge led to significant increase in the infiltration of inflammatory cells into the peribronchiolar tissues (*P* < 0.001; Fig. 3). A pronounced increase in the level of mucus secretion and marked thickening of the basement-membrane were also observed in the bronchi of the OVA-induced asthmatic mice compared with the mice in the NC group. A reduction in inflammatory infiltrates was observed in the mice treated with the *Loki zupa* decoction and its extracts, as well as the DEX treatment groups, compared with mice in the A group (*P* < 0.001). The LN and LE treatment groups were more effective in terms of their ability to alleviate inflammation compared with the L 7.0, L 3.5 and LW treatment groups (*P* < 0.001), and no significant differences were observed for the DEX treatment group (*P* = 0.062, *P* = 0.223).

### *Loki zupa* decoction and its extracts treatment inhibited inflammatory cells in BALF of OVA-induced asthmatic mice

**Fig. 4:**
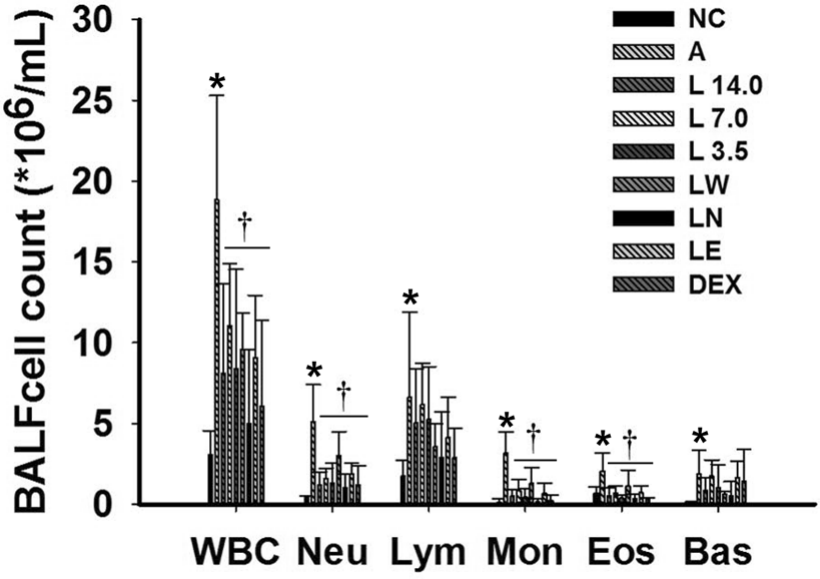
*Loki zupa* decoction and its extracts inhibited the inflammatory cells in BALF. The total white blood cell (WBC), neutrophil (Neu), lymphocyte (Lym), monocyte (Mon), eosinophil (Eos) and basocyte (Bas) counts in BALF were analyzed and the resulting data expressed as the mean ± SD. * *P* < 0.05 *vs.* NC group, ^†^ *P*< 0.05 *vs.* A group

The total white blood cell (WBC), neutrophil (Neu), lymphocyte (Lym), monocyte (Mon), eosinophil (Eos) and basocyte (Bas) counts in the BALF samples were analyzed to investigate the effects of the *Loki zupa* decoction and its extracts on the inflammatory cells in the lung tissue. OVA inhalation led to significant increases in the total WBC, Neu, Lym, Mon, Eos and Bas counts in the BALF samples (*P* = 0.001, *P* < 0.001, *P* = 0.047, *P* < 0.001, *P* = 0.007, *P* = 0.041; Fig. 4). Treatment with *Loki zupa* decoction and its extracts, as well as DEX, led to a decrease in the total WBC (*P* = 0.013, *P* = 0.044, *P* = 0.009, *P* = 0.029, *P* = 0.001, *P* = 0.014, *P* = 0.004), Neu (*P* = 0.001, *P* = 0.001, *P* = 0.001, *P* = 0.047, *P* < 0.001, *P* = 0.003, *P* = 0.001), Mon (*P* < 0.001, *P* < 0.001, *P* < 0.001, *P* = 0.001, *P* < 0.001, *P* < 0.001, *P* < 0.001) and Eos (*P* = 0.003, *P* = 0.004, *P* = 0.001, *P* = 0.047, *P* < 0.001, *P* = 0.004, *P* = 0.001) counts in the BALF samples. However, treatment with the *Loki zupa* decoction and DEX did not led to a decrease in the Lym (*P* = 0.519, *P* = 0.848, *P* = 0.543, *P* = 0.212, *P* = 0.089, *P* = 0.273, *P* = 0.133) or Bas (*P* = 0.309, *P* = 0.860, *P* = 0.337, *P* = 0.209, *P* = 0.123, *P* = 0.807, *P* = 0.631) counts in the BALF samples collected from the OVA-induced asthmatic mice.

### *Loki zupa* decoction and its extracts treatment regulated inflammatory cytokines in BALF and serum of OVA-induced asthmatic mice

**Fig. 5:**
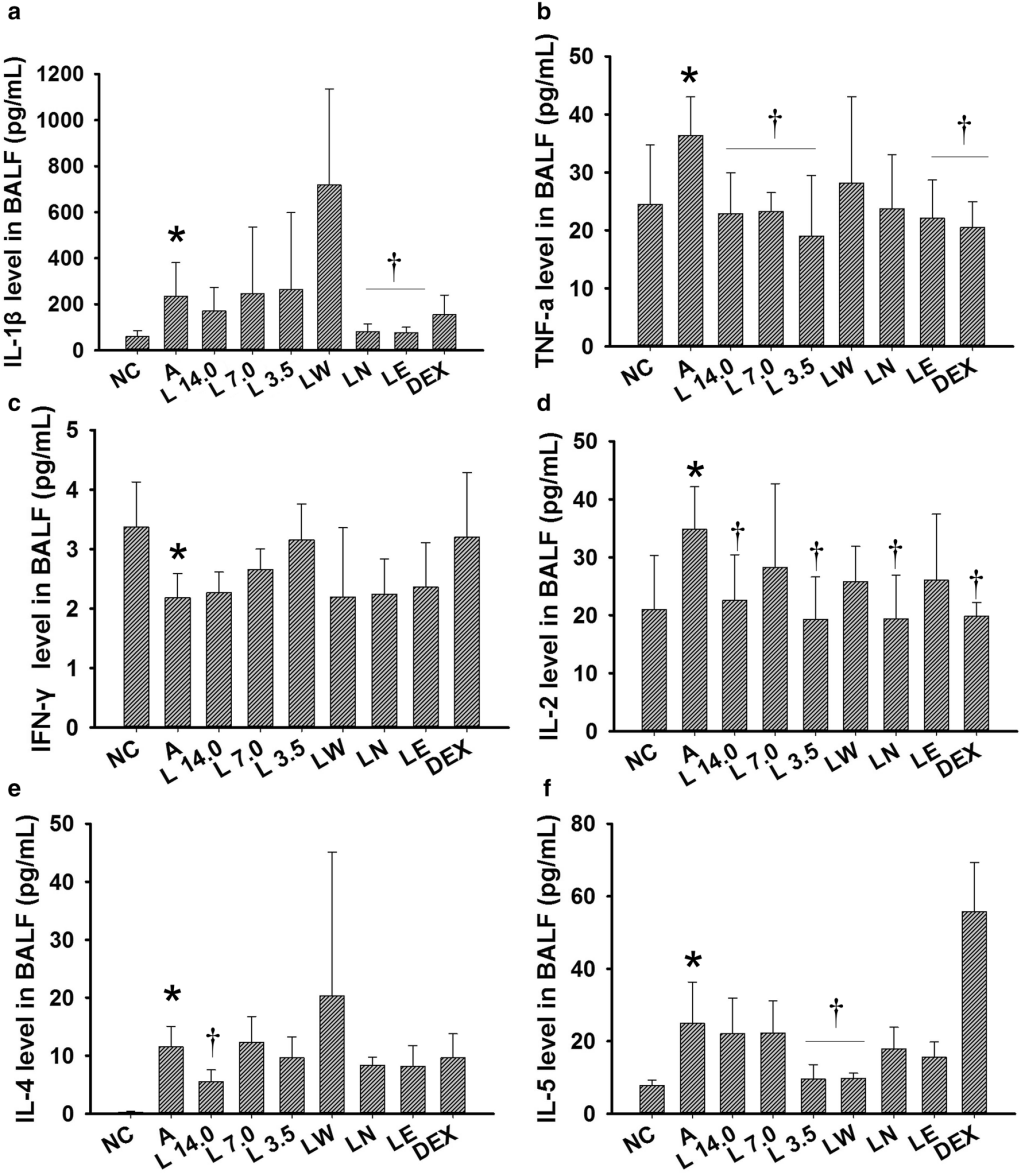
*Loki zupa* decoction and its extracts regulated the secretion of several inflammatory cytokines in BALF. The IL-1β, TNF-α, IFN-γ, IL-2, IL-4 and IL-5 levels in BALF were measured by Bio-Plex and the resulting data expressed as mean ± SD. **a** IL-1β level in BALF, **b** TNF-α level in BALF, **c** IFN-γ level in BALF, **d** IL-2 level in BALF, **e** IL-4 level in BALF, **f** IL-5 level in BALF. *P < 0.05 *vs.* NC group, ^†^ *P* < 0.05 *vs.* A group

**Fig. 6:**
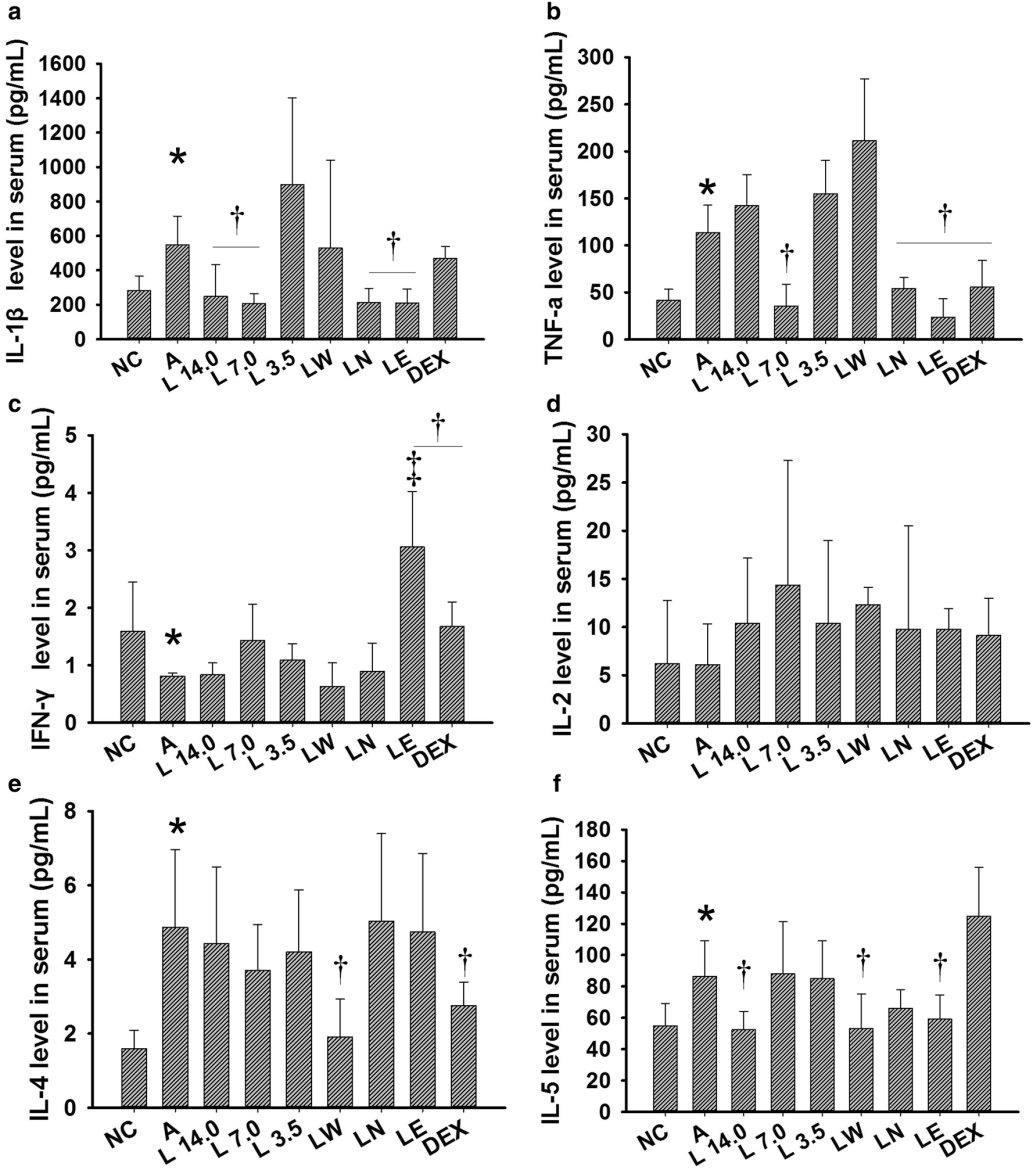
*Loki zupa* decoction and its extracts regulated the secretion of several inflammatory cytokines in serum. The IL-1β, TNF-α, IFN-γ, IL-2, IL-4 and IL-5 levels in serum were measured by Bio-Plex and the resulting data expressed as mean ± SD. **a** IL-1β level in serum, **b** TNF-α level in serum, **c** IFN-γ level in serum, **d** IL-2 level in serum, **e** IL-4 level in serum, **f** IL-5 level in serum. *P < 0.05 *vs.* NC group,^†^ *P* < 0.05 *vs.* A group, ^‡^ *P* < 0.05 *vs.* DEX group

Marked increases were observed in the IL-1β (Fig. 5a, *P* = 0.042), TNF-α (Fig. 5b,*P* = 0.045), IL-4 (Fig. 5e, *P* = 0.038) and IL-5 (Fig. 5f, *P* = 0.002) levels in the BALF (Fig. 5) and serum (Fig. 6a, *P* = 0.045, Fig. 6b, *P* = 0.042, ​Fig. 6e, *P* = 0.008, ​Fig. 6f, *P* = 0.047) (Fig. 6) samples collected from the OVA-induced asthmatic mice, as well as a decrease in their IFN-γ levels (​Fig. 5c, *P* = 0.039, ​Fig. 6c, *P* = 0.038). Treatment with the *Loki zupa* decoction or its extracts led to a reduction in the levels of IL-1β, TNF-α, IL-4 and IL-5 in the BALF and serum samples, as well as an increase in IFN-γ levels (Fig. 5 or Fig. 6).

The LN and LE treatment groups showed reduced levels of IL-1β in their BALF samples (*P* = 0.039, *P* = 0.042; Fig. 5a) compared with the OVA-induced asthma mice. However, DEX was found to be ineffective for lowering the IL-1β levels in BALF (*P* = 0.466; Fig. 5a). The three different doses of *Loki zupa* decoction used in the current study, as well as the LE and DEX treatment groups showed dramatic decreases in the TNF-α levels found in their BALF samples (*P* = 0.037, *P* = 0.043, *P* = 0.009, *P* = 0.028, *P* = 0.016; Fig. 5b) compared with A group. The L 3.5 and DEX treatment groups showed a tendency towards increased IFN-γ levels in their BALF samples, although these results were not statistically different (*P* = 0.247, *P* = 0.173; Fig. 5c). The L 14.0, L 3.5, LN and DEX treatment groups showed reduced IL-2 levels in their BALF sampled (*P* = 0.049, *P* = 0.019, *P* = 0.019, *P* = 0.023; Fig. 5d) compared with A group. The L 14.0 treatment group showed a decrease in the IL-4 levels in its BALF sampled (*P* = 0.047; Fig. 5e) compared with A group. Furthermore, the L 3.5 and LW treatment groups showed reduced levels of IL-5 compared with the OVA-induced asthmatic mice (*P* = 0.004, *P* = 0.004; Fig. 5f). However, treatment with DEX had no effect on the IL-4 and IL-5 levels in the BALF samples of the mice in A group.

The L 14.0, L 7.0, LN and LE treatment groups showed significant decreases in the IL-1β levels observed in the serum samples taken from the OVA-induced asthmatic mice (*P* = 0.044, *P* = 0.045, *P* = 0.049, *P* = 0.047; Fig. 6a) compared with mice in A group. However, DEX treatment did not result in a significant decrease in IL-1β levels in the serum (*P* = 0.63; Fig. 6a). The L 7.0, LN, LE and DEX treatment groups showed significant decreases in the TNF-α levels of the serum samples collected from the asthmatic mice (*P* = 0.001, *P* = 0.01, *P* < 0.001, *P* = 0.012; Fig. 6b) compared with the mice from the untreated group. The LE and DEX treatment groups showed a significant increase in the IFN-γ levels of the serum samples compared with the OVA-induced asthmatic mice (*P* < 0.001, *P* = 0.03; Fig. 6c). Moreover, LE treatment was found to be more effective than DEX for promoting the levels of IFN-γ in serum (*P* = 0.001). None of the treatment groups tested in the current study had a discernible impact on the levels of IL-2 secretion in the serum (Fig. 6d). However, the LW and DEX treatment groups showed a marked decrease in the IL-4 levels of the serum (*P* = 0.021, *P* = 0.049; Fig. 6e) compared with A group. Lastly, the serum samples collected from the L 14.0, LW and LE treatment groups showed significant reductions in the secretion of IL-5 (*P* = 0.036, *P* = 0.04, *P* = 0.049; Fig. 6f) compared with the untreated mice. However, DEX exhibited no inhibitory activity towards the IL-5 levels in serum.

### Treatment with *Loki zupa* decoction or its extracts regulated mRNA expression of Th2 and Th1 cytokines in OVA-induced asthmatic mice

**Fig. 7:**
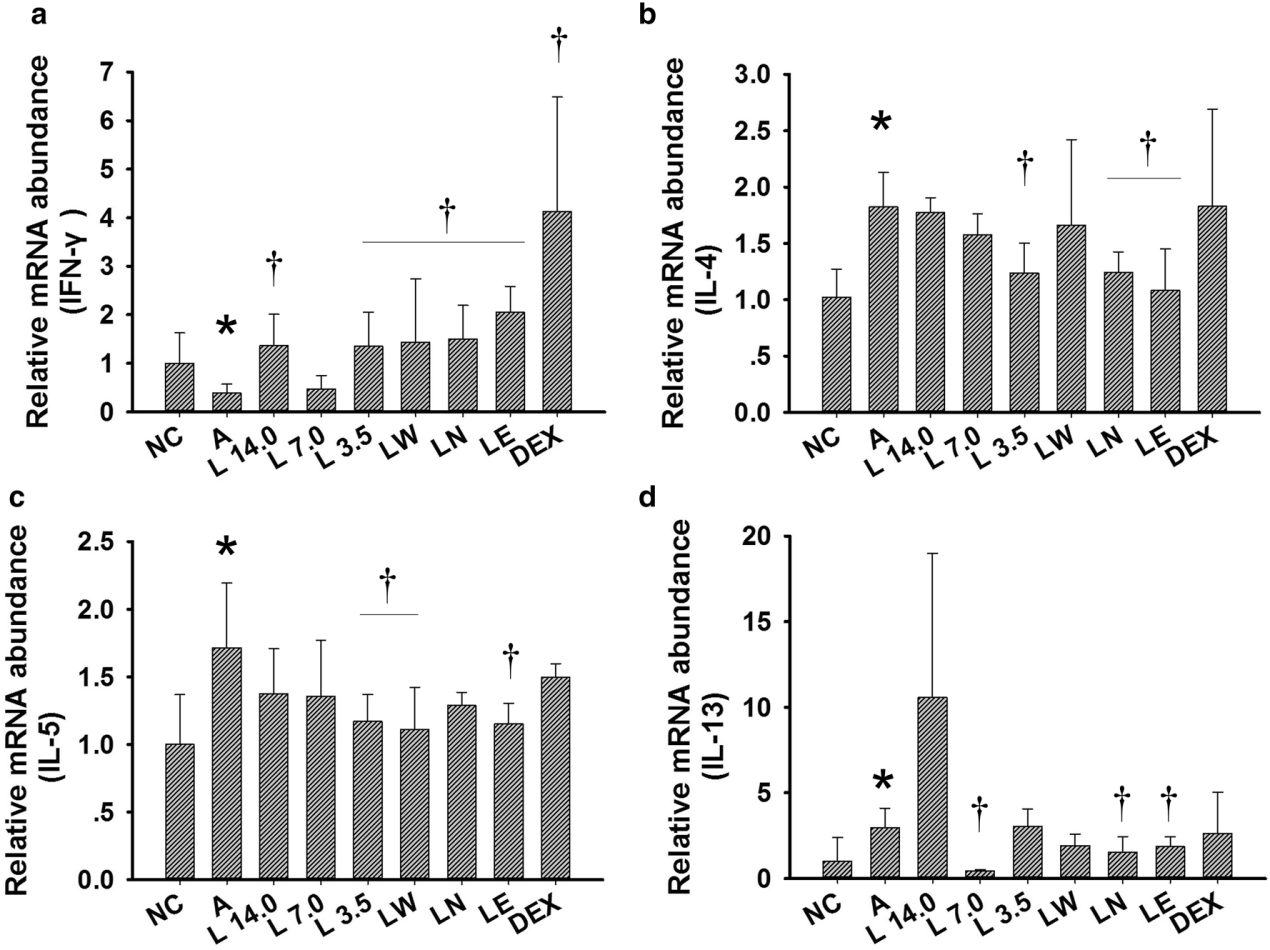
*Loki zupa* decoction and its extracts inhibited and promoted the Th2 and Th1 cytokine mRNA expression levels of asthmatic mice, respectively. The mRNA expression levels of IL-4, IL-5, IL-13 and IFN-γ in the lung tissues were measured by qPCR and the data expressed as the mean ± SD. **a** mRNA expression level of IFN-γ, **b** mRNA expression level of IL-4, **c** mRNA expression level of IL-5, **d** mRNA expression level of IL-13. *P < 0.05 *vs.* NC group, ^†^ *P* < 0.05 *vs.* A group

OVA inhalation led to enhanced levels of IL-4 (Fig. 7b), IL-5 (Fig. 7c) and IL-13 (Fig. 7d) mRNA expression, as well as reduced levels of IFN-γ (Fig. 7a) mRNA expression in the OVA-induced asthmatic mice compared with mice in the NC group (*P* < 0.001, *P* = 0.045, *P* = 0.049, *P* = 0.047; Fig. 7). Treatment with the *Loki zupa* decoction or its extracts led to reduced IL-4 (Fig. 7b) , IL-5 (Fig. 7c) and IL-13 (Fig. 7d) mRNA expression levels in asthmatic mice, as well as improved IFN-γ(Fig 7a)  mRNA expression. With the exception of the L 7.0 group, all of the other treatment groups exhibited a significant promotion effect towards the mRNA expression of IFN-γ (*P* = 0.039, *P* = 0.04, *P* = 0.048, *P* = 0.035, *P* < 0.001, *P* < 0.001; Fig. 7a). The L 3.5, LN and LE treatment groups also showed a dramatic decrease in the mRNA expression of IL-4 (*P* = 0.021, *P* = 0.023, *P* = 0.017; Fig. 7b). Furthermore, mice in the L 3.5, LW and LE treatment groups demonstrated a marked reduction in their IL-5 mRNA expression (*P* = 0.043, *P* = 0.044, *P* = 0.04; Fig. 7c). The L 7.0, LN and LE treatment groups led to an obvious decrease in the mRNA expression of IL-13 (*P* < 0.001, *P* = 0.037, *P* = 0.046; Fig. 7d). DEX treatment had no effect on the mRNA expression levels of IL-4, IL-5 and IL-13 in our study (*P* = 0.91, *P* = 0.83, *P* = 0.792).

## Discussion

Several characteristic features of acute asthma are observed in the OVA-induced mice asthma model, including an exaggerated airway response to Mch and eosinophil-rich airway inflammation [19]. Short-term OVA inhalation resulted in a marked increase in R_L_, as well as a pronounced decrease in Cdyn towards increasing doses of Mch. H&E staining of the lung slices revealed that there was a significant increase in the infiltration of Eos into the airways of the asthmatic mice, as well as the levels of mucus secretion. Treatment with *Loki zupa* decoction led to a dramatic decrease in the elevated AHR towards progressive Mch doses in asthmatic mice. Furthermore, the L 3.5 and LE treatment groups showed a reduction in R_L_, which was almost identical to that of DEX, highlighting the potential value of these materials for the treatment of asthma. Treatment withthe *Loki zupa* decoction resulted in a decrease in the number of WBC, Neu, Eos and Mon cell counts in the lungs of the asthmatic mice. There was a direct correlation between Eos counts and asthma symptom scores [20]. According to the results of several previous studies [21, 22], treatments aimed at reducing airway eosinophilia numbers are effective for controlling asthma symptoms. Treatment with the *Loki zupa* decoction also had a direct impact on Eos numbers in the airways, highlighting its efficacy for controlling asthma.

In our study, *Loki zupa* decoction exerted prominent inhibitory effects towards the levels of IL-1β and TNF-α. Elevated Th2 cell levels and cytokine functions are characteristic features of allergic asthma, which have been implicated in eosinophilic inflammation as well as AHR [5, 23, 24]. The administration of L 14.0 and LW resulted in a dramatic reduction in IL-4 levels in the BALF and serum samples collected from the asthmatic mice. IL-4 is essential for the development of AHR in murine models [25, 26], indicating that the decrease observed in AHR following the treatment of the mice with *Loki zupa* decoction could be attributed to a reduction in IL-4 levels. IL-5 is involved in Eos maturation, differentiation, recruitment, and survival [6]. L 3.5 and LW, as well as L 14.0 and LE inhibited the OVA-induced increase in IL-5 levels in the BALF and serum samples collected from the mice. However, DEX had no effect on the levels of IL-4 and IL-5 in the BALF samples or the IL-5 levels in the serum samples. Th2 blockade is effective for suppressing the features of allergic disease in mice [27]. Anti-IL-5 antibody has therefore been reported to reduce the risk of exacerbation in patients with severe eosinophilic asthma [28]. Approximately 50 % of asthmatic patients have a Th2-driven phenotype [29]. The treatment of the mice with *Loki zupa* decoction inhibited the expression of the Th2 cytokines, indicating that its related anti-asthmatic effect could be attributed to its ability to inhibit the activity of the Th2 cells. Treatment with *Loki zupa* decoction or its extracts also led to a significant down-regulatory effect on the gene amplification levels of IL-4, IL-5 and IL-13. However, DEX treatment had no discernible impact on the levels of these cytokines. IL-13 is a key Th2 cytokine that might be involved in the development of asthma [30, 31]. Transgenic mice over-expressing IL-13 in their lung develop some of the features of airway inflammation typically observed in asthma [32]. Treatment with L 7.0, LN and LE led to a remarkable decrease in the mRNA amplification of IL-13, indicating that the anti-inflammatory effects of these materials could be related to their ability to inhibit the expression of this cytokine.

Th1 cells have been reported to exhibit inhibitory effects towards the inflammation of asthmatic airways by inhibiting Th2 cytokine responses [33]. Furthermore, there are generally lower levels ofthe Th1 cytokine IFN-γ in asthma [34, 35]. The treatment of the mice with *Loki zupa* decoction or its extracts and DEX effectively promoted the expression of IFN-γ at the gene and protein levels. This result therefore indicated that the anti-inflammatory activity of *Loki zupa* decoction towards asthma could be attributed to its ability to promote the activity of Th1 cells, as well as its antagonistic potential towards the function of Th2 cells. IFN-γ has been shown to inhibit antigen-induced AHR in mice [36], indicating that elevated levels of IFN-γ might be involved in the responses observed in the *Loki zupa* decoction-treated groups.

Except for the decrease observed in the serum level of IL-4, DEX promoted the expression of the Th1 cytokine IFN-γ in the serum and lung tissue samples, but did not lead to a decrease in the expression levels of IL-4 and IL-5 in BALF. In contrast, *Loki zupa* decoction exhibited inhibitory activity towards the expression of IL-4 and IL-5 in the BALF and serum samples, as well as the lung tissues. However, *Loki zupa* decoction also exhibited a significant promotion effect towards IFN-γ, indicating that the anti-inflammatory effect of the *Loki zupa* decoction treatment could be attributed to the promotion and suppression of the Th1 and Th2 cytokine expression levels, respectively. This promotion of the Th1 cell function could therefore be responsible for the effect of DEX on asthma.

The JAK-STAT pathway promotes the differentiation of naive T cells into Th2 cells, as well as inducing the secretion of Th2 cytokines [37–39]. STAT4 has been implicated in IL-12-dependent Th1 cellular responses, and mice lacking STAT4 have been reported to show severe deficiencies in mounting a Th1 response [40]. Furthermore, IL-4-induced STAT6 activation has been reported to contribute to the differentiation of Th2 cells [41]. The JAK-STAT pathway could therefore be involved in regulating the Th1/Th2 imbalance observed in asthma patients following their treatment with *Loki zupa* decoction.

## Conclusion

The treatment of mice with *Loki zupa* decoction and its extracts resulted in a decrease in AHR and attenuated airway inflammation in an OVA-induced asthma mouse model by promoting and suppressing the cellular functions of Th1 and Th2, respectively.

## Additional files

10.1186/s13020-016-0094-9 Ethical approval document
10.1186/s13020-016-0094-9 The ARRIVE Guidelines Checklist.

## Abbreviations

OVA: ovalbumin
AHR: airway hyperresponsiveness
Mch: methacholine
NC: normal control
A: asthma
*Loki zupa* decoction 14.0 g/kg: L 14.0
*Loki zupa* decoction 7.0 g/kg: L 7.0
*Loki zupa* decoction 3.5 g/kg: L 3.5
H&E: hematoxylin and eosin
BALF: bronchoalveolar lavage fluid
LW: water extract
LE: ethyl acetate extract
LN: *n*-butanol extract
DEX: dexamethasone
R_L_: airway resistance
Cdyn: lung dynamic compliance
WBC: white blood cell
Neu: neutrophil
Mon: monocyte
Eos: eosinophil
Lym: lymphocyte
Bas: basocyte

## References

[1] van Schayck OC (2013). Global strategies for reducing the burden from asthma. Prim Care Respir J..

[2] Saglani S, Lloyd CM (2015). Novel concepts in airway inflammation and remodelling in asthma. Eur Respir J.

[3] Tourangeau LM, Kavanaugh A, Wasserman SI (2011). The role of monoclonal antibodies in the treatment of severe asthma. Ther Adv Respir Dis.

[4] Caramori G, Ito K, Adcock IM (2004). Targeting Th2 cells in asthmatic airways. Curr Drug Targets Inflamm Allergy.

[5] Lloyd CM, Saglani S (2013). T cells in asthma: influences of genetics, environment, and T-cell plasticity. J Allergy Clin Immunol.

[6] Shalaby KH, Martin JG (2010). Overview of asthma; the place of the T cell. Curr Opin Pharmacol.

[7] Barrett NA, Austen KF (2009). Innate cells and T helper 2 cell immunity in airway inflammation. Immunity.

[8] Diamant Z, Tufvesson E, Bjermer L (2013). Which biomarkers are effective for identifying Th2-driven inflammation in asthma. Curr Allergy Asthma Rep..

[9] Fajt ML, Gelhaus SL, Freeman B, Uvalle CE, Trudeau JB, Holguin F, Wenzel SE (2013). Prostaglandin D(2) pathway upregulation: relation to asthma severity, control, and TH2 inflammation. J Allergy Clin Immunol.

[10] Lin WH, Wu CR, Lee HZ, Kuo YH, Wen HS, Lin TY, Lee CY, Huang SY, Lin CY (2013). Induced apoptosis of Th2 lymphocytes and inhibition of airway hyperresponsiveness and inflammation by combined lactic acid bacteria treatment. Int Immunopharmacol.

[11] Nagai H (2012). Recent research and developmental strategy of anti-asthma drugs. Pharmacol Ther.

[12] Finotto S, Neurath MF, Glickman JN, Qin S, Lehr HA, Green FH, Ackerman K, Haley K, Galle PR, Szabo SJ (2002). Development of spontaneous airway changes consistent with human asthma in mice lacking T-bet. Science.

[13] Ekim M, Rozi N, Yusup A (2011). Effect of Uighur medicine *Hyssopus officinalis* L ethyl aectate on inflammatory response in asthma rats. Ke ji dao bao..

[14] Hou M, Zhu M, Ma XM, Ding JB, Ma J, Ma XP, Wang XX, Wang YN. Effect of Uighur Medicine *Hyssopus officinalis* L. on serum IL-17 level and balance of Th1/Th2 of asthma rats. *Zhongguo xian dai yi xue* 2010,03(02):365-368.

[15] Zdolsek HA, Janefjord CK, Falth-Magnusson K, Jenmalm MC (2003). Reduced IL-2-induced IL-12 responsiveness in atopic children. Pediatr Allergy Immunol..

[16] Xu F, Zhao J, He J, Chen Y, Tan W (2012). Pharmacognostic identification of Uygur medicine *Hyssopus cuspidatus* and its adulterant *Nepeta bracteata*. Zhongguo yao fang.

[17] Yang XJ, Yang WJ (2011). Quality Standard of Xinjiang Uygur medicine *Iris halophila*. Zhongguo yao fang.

[18] Jin H, Luo Q, Zheng Y, Nurahmat M, Wu J, Li B, Lv Y, Wang G, Duan X, Dong J (2013). CD4 + CD25 + Foxp3 + T cells contribute to the antiasthmatic effects of Astragalus membranaceus extract in a rat model of asthma. Int Immunopharmacol.

[19] Reddy AT, Lakshmi SP, Reddy RC (2012). Murine model of allergen induced asthma. J Vis Exp JoVE.

[20] Nair P (2013). What is an “eosinophilic phenotype” of asthma?. J Allergy Clin Immunol.

[21] Steinke JW, Negri J, Liu L, Payne SC, Borish L (2014). Aspirin activation of eosinophils and mast cells: implications in the pathogenesis of aspirin-exacerbated respiratory disease. J Immunol.

[22] Fulkerson PC, Rothenberg ME (2013). Targeting eosinophils in allergy, inflammation and beyond. Nat Rev Drug Discov.

[23] Hams E, Fallon PG (2012). Innate type 2 cells and asthma. Curr Opin Pharmacol.

[24] Corrigan C (2012). Mechanisms of asthma. Medicine.

[25] Perkins C, Yanase N, Smulian G, Gildea L, Orekov T, Potter C, Brombacher F, Aronow B, Wills-Karp M, Finkelman FD (2011). Selective stimulation of IL-4 receptor on smooth muscle induces airway hyperresponsiveness in mice. J Exp Med.

[26] Tanaka H, Nagai H, Maeda Y (1998). Effect of anti-IL-4 and anti-IL-5 antibodies on allergic airway hyperresponsiveness in mice. Life Sci..

[27] Finkelman FD, Hogan SP, Hershey GK, Rothenberg ME, Wills-Karp M (2010). Importance of cytokines in murine allergic airway disease and human asthma. J Immunol.

[28] Pavord ID, Korn S, Howarth P, Bleecker ER, Buhl R, Keene ON, Ortega H, Chanez P (2012). Mepolizumab for severe eosinophilic asthma (DREAM): a multicentre, double-blind, placebo-controlled trial. Lancet.

[29] Apte K, Salvi S (2015). Vitamin D reduces eosinophilic airway inflammation in nonatopic asthma: are we sure?. J Allergy Clin Immunol.

[30] Ingram JL, Kraft M (2012). IL-13 in asthma and allergic disease: asthma phenotypes and targeted therapies. J Allergy Clin Immunol..

[31] Elias JA, Lee CG (2011). IL-13 in asthma. The successful integration of lessons from mice and humans. Am J Respir Crit Care Med.

[32] Qaseem AS, Sonar S, Mahajan L, Madan T, Sorensen GL, Shamji MH, Kishore U (2013). Linking surfactant protein SP-D and IL-13: implications in asthma and allergy. Mol Immunol.

[33] Kips JC, Brusselle GJ, Joos GF, Peleman RA, Tavernier JH, Devos RR, Pauwels RA (1996). Interleukin-12 inhibits antigen-induced airway hyperresponsiveness in mice. Mol Immunol.

[34] Brooks GD, Buchta KA, Swenson CA, Gern JE, Busse WW (2003). Rhinovirus-induced interferon-gamma and airway responsiveness in asthma. Mol Immunol.

[35] Akpinarli A, Guc D, Kalayci O, Yigitbas E, Ozon A (2002). Increased interleukin-4 and decreased interferon gamma production in children with asthma: function of atopy or asthma?. J Asthma..

[36] Yoshida M, Leigh R, Matsumoto K, Wattie J, Ellis R, O’Byrne PM, Inman MD (2002). Effect of interferon-gamma on allergic airway responses in interferon-gamma-deficient mice. Am J Respir Crit Care Med.

[37] Espinosa K, Bosse Y, Stankova J, Rola-Pleszczynski M (2003). CysLT1 receptor upregulation by TGF-beta and IL-13 is associated with bronchial smooth muscle cell proliferation in response to LTD4. J Allergy Clin Immunol.

[38] Matsuse H, Kong X, Hu J, Wolf SF, Lockey RF, Mohapatra SS (2003). Intranasal IL-12 produces discreet pulmonary and systemic effects on allergic inflammation and airway reactivity. Int Immunopharmacol.

[39] Ashino S, Takeda K, Li H, Taylor V, Joetham A, Pine PR, Gelfand EW (2014). Janus kinase 1/3 signaling pathways are key initiators of TH2 differentiation and lung allergic responses. J Allergy Clin Immunol.

[40] Shirai T, Suzuki K, Inui N, Suda T, Chida K, Nakamura H (2003). Th1/Th2 profile in peripheral blood in atopic cough and atopic asthma. Clin Exp Allergy.

[41] Gao Z, Kang Y, Xu Y, Shang Y, Gai J, He Q (2002). Inhibition of allergic responsiveness in a murine asthma model via IFN-gamma transgene expression. Chin Med J.

