# Supplementary material for: Loki zupa (Luooukezupa) decoction reduced airway inflammation in an OVA-induced asthma mouse model
Source: Chin Med. 2016 Apr 29;11:22. doi: 10.1186/s13020-016-0094-9 (PMC4851804; doi:10.1186/s13020-016-0094-9)
Supplement: Supplementary file 1 — 10.1186/s13020-016-0094-9 Ethical approval document [file 13020_2016_94_MOESM1_ESM.pdf]

复旦大学药学院实验动物伦理委员会

研究项目伦理审批件

伦药批第（2013-06-HSYY-DJC-02）号

<table>
<tr><td>项目名称</td><td colspan="5">罗欧克祖帕对 OVA 诱导的哮喘小鼠气道炎症的调节作用研究</td></tr>
<tr><td>项目类别</td><td colspan="5">基础 √ 临床____ 药物____</td></tr>
<tr><td>项目来源</td><td colspan="3">国家 973 课题</td><td>经 费</td><td>1100 万元</td></tr>
<tr><td>申办单位</td><td colspan="3">复旦大学附属华山医院中西医结合科</td><td>主要负责人</td><td>魏颖</td></tr>
<tr><td>研究部门</td><td>复旦大学附属华山医院中西医结合科</td><td>项目负责人</td><td>董竞成</td><td>职 称</td><td>教授
主任医师</td></tr>
<tr><td colspan="6">伦理审查意见</td></tr>
<tr><td colspan="4">Δ 同意</td><td colspan="2">√</td></tr>
<tr><td colspan="4">Δ 修改后同意</td><td colspan="2"></td></tr>
<tr><td colspan="4">Δ 不同意（项目终止或暂停）</td><td colspan="2"></td></tr>
<tr><td colspan="6">审批意见

同意实施

主任委员（签名）[illegible]

复旦大学药学院实验动物伦理委员会
（盖章）

2013 年 06 月 03 日</td></tr>
</table>

复旦大学药学院动物实验申请书

一、实验项目

项目名称： 罗欧克祖帕对 OVA 诱导的哮喘小鼠气道炎症的调节作用研究

项目编号：2009CB523000

经费来源： 国家 973 课题

项目起止日期： 2013 年 6 月至 2013 年 8 月

动物实验地点： 复旦大学（药学院）　　环境设施许可证号：SYXK(沪)2010-0099

二、项目负责人：

姓　　名 董竞成　　职　　称 教授，主任医师

单　　位 复旦大学附属华山医院　　联系电话 13601761761

三、负责动物实验执行人

姓　名	职　称	单　　位	联系电话	动物实验经验	动物实验岗位证书编号
包燕	技术员	复旦大学药学院	51980014	有	11114549
钱梦婷	技术员	复旦大学药学院	51980014	有	11114546

四、实验所需动物

类　别	品　种	数量	来　　源	用途（见备注）
小鼠	Balb/c	108	上海西普尔-必凯实验动物有限公司	①

备注：用途　①医学研究　②药物疫苗　③健康食品　④农业研究　⑤教学训练　⑥其他

五、动物饲养管理：　√由动物中心专人负责

□由实验室人员负责。

六、简述实验中所进行动物试验内容、方法、剂量与步骤（包括动物的给药、注射、麻醉、手术及术后照顾等），并说明使动物痛苦降至最低的方法。

108 只雌性 Balb/c 小鼠随机分为 9 组，分别为急性正常对照组、急性哮喘模型组、罗欧克祖帕 14g/kg 组、罗欧克祖帕 7g/kg 组、罗欧克祖帕 3.5 g/kg 组、水洗组（相当于 7g/kg 药材）、正丁醇组（相当于 7g/kg 药材）、乙酸乙酯组（相当于 7g/kg 药材）；地塞米松 1mg/kg 组，每组 12 只，采用 OVA 致敏（d_0腹腔注射含 OVA 20ug 和氢氧化铝 2mg 的混悬液 0.2ml 致敏，d_7 、d_{14}、 d_{21}重复致敏三次）和激发（d_{25}以 3%（3g OVA 溶解于 100ml 无菌生理盐水）的卵蛋白生理盐水溶液 5L/min 雾化吸入（使用医用超声雾化器）30 分钟）建立哮喘小鼠模型，用 OVA 连续激发 7 天建立急性哮喘小鼠模型；每次激发前 1 小时腹腔注射给药（0.3ml/只），最后一次激发 24 小时后，2%的戊巴比妥钠腹腔注射（70mg/Kg）麻醉后测定气道高反应性，然后取血，取肺泡灌洗液及肺组织。

七、实验结束后动物的处置方式（包括复原处置、安乐死及尸体处理方式）。

本实验取材后动物即死亡，动物尸体用专门塑料袋包装后，放入指定动物尸体暂存冰柜，由动物中心集中处理。

八、简述申请动物实验方案合理性。

根据实验目的、统计学方法，一般小型动物 8-10 只，比如小鼠，估计样本含量的公式计算方法参考专业文献：【医学科研方法学】，主编：梁万年，出版社：人民卫生出版社。

在前期研究的基础上，进行合理方案设计，优化实验过程，同时，实验人员技术熟练，实验过程中避免增加实验动物的痛苦。

申请人保证以上所填资料完全属实，并确认此申请的执行与运作符合《实验动物管理条例》和国家有关法律、法规。

申请人签名：[signature]　　　　单位主管签名：

单位盖章：　　　　日期：

评审结果

☑通过
☐改善后复审
☐不通过
改善或者不通过的意见：

评审人：[signature]　　　　日期：
伦理委员会签章：　　　　日期：
